# Care and choice architecture: Relatives’ support for adults with intellectual disabilities in supported decision-making processes

**DOI:** 10.1177/17446295241297184

**Published:** 2024-10-30

**Authors:** Sadeta Demic, Rosemarie van den Breemer, Halvor Hanisch, Inger Marie Lid

**Affiliations:** 87446 VID Specialized University, Norway; Inland School of Business and Social Sciences, Norway; 87446 VID Specialized University, Norway; 87446 VID Specialized University, Norway

**Keywords:** choice architecture, CRPD, ethics of care, intellectual disability, relatives’ experiences, supported decision making

## Abstract

The UN Convention on the Rights of Persons with Disabilities (CRPD) describes supported decision making as a fundamental human right. This study explores relatives’ experiences, dilemmas, as well as key factors in supported decision-making processes with adults with intellectual disabilities living in municipal housing. The study draws on qualitative, in-depth interviews with relatives. Findings suggest that we can learn about supported decision making by using choice architecture and care theories, demonstrating that: (a) supported decision-making sometimes requires careful facilitation with a focus on the person's preferences/perspective; this careful facilitation can be understood in terms of choice architecture, (b) choice architecture in the context of intellectual disability requires an intentionality of care and a focus on the person’s preferences/perspective, (c) relatives are concerned, not only with support in the moment, but also the effect of the decision in the long term, and (d) relatives’ care is a significant factor in meeting choice architecture in systemic conditions.

## Introduction

The UN Convention on the Rights of Persons with Disabilities (CRPD) defines self-determination (Article 12.2) as the right to make one’s own decisions. The same document describes supported decision making (Article 12.3) as the right to receive the support one needs to be able to make one’s own decisions (UN, 2006). While the treaty was internationally drafted and signed in 2006 and 2007 respectively, it was first ratified in Norway in 2013. Although the convention is not incorporated into Norwegian law, political guidelines in Norway point to supported decision making (SDM) and self-determination as a fundamental and guiding principle in care work (Helsedirektoratet (Directorate of Health) 2021; NOU 2019: 14).

Research shows no uniform definition of supported decision making (Then et al., 2018). Common to the research literature on SDM is that the person’s own will and preferences should govern decision-making processes and that the person who provides decision-making support should know the person they are supporting well (Bigby and Douglas, 2020; Watson et al., 2017). In Norway, SDM is defined as ‘any process that enables the person to make their own decisions and/or to express their own will and preferences’ (Helsedirektoratet, 2021). The term supported decision making refers to everyday support processes where an individual receives support from a support person who collects and adapts information to support the person in being able to make their own decisions and exercising their autonomy. Using different strategies in such practical everyday decision support processes takes place in many relationships and ‘can be implemented independently of legislation’ (Then et al., 2018: 64). Although certain decision support processes (such as the conclusion of agreements) are described as formal SDM, we are concerned with informal SDM in this study. Informal SDM is all support that takes place in everyday life and is provided by family, friends, or service providers (Bigby and Douglas, 2020).

This article focuses on relatives, in this case immediate family, of persons with intellectual disability, since relatives as well as service providers help persons with intellectual disability understand the situations and choices that they face (Blanck and Martinis 2015). Family members often have a distinctive role because of their intimate and long-term knowledge of the person. Studies suggest that familiarity with the person, commitment, knowledge of human rights, and support of a person’s attitudes and skills are fundamental in SDM processes (Bigby and Douglas, 2020; Bigby et al., 2019; Demic et al., 2023; Demic and Gjermestad, 2021). However, few studies have researched relatives’ experiences.

Relatives’ experiences can help expand our knowledge about SDM for persons with intellectual disability. However, the intimate and tacit knowledge of relatives can also stand in the way of trying out new options, for example because the relatives do not see these in line with the person’s preferences. Support persons, relatives, and service providers can strengthen a person’s capacity for self-determination and individual freedom, but also lead to paternalism (Bigby and Douglas, 2020). Therefore, as research shows, good relationships are important (Agran et al., 2010; Knox et al., 2017), including trust, positive attitudes toward risk, and a commitment to maintaining an individual’s rights (Agran et al., 2010). Studies suggest that a lack of encouragement and support for adults with intellectual disability can lead to limited awareness of their life options and preferences, as well as a passive and limited life (Curryer et al., 2015; Skarstad, 2023). Allowing for the continual exploration of an individual’s preferences by encouraging and trying out new activities, we are certain to promote the individual’s life, simultaneously as we can provide carer’s with better knowledge of who the individual is as a person (Vehmas and Mietola, 2021). Protecting adults with intellectual disability from risk and bad decisions can also inhibit opportunities for learning (Bowey and McGlaughlin, 2005; Wiesel et al., 2021). Therefore, for SDM processes to be successful, support persons must cooperate with the individual in order to recognize advantages and disadvantages and learn to balance risks and benefits (Seale et al., 2013).

This study aimed to explore the relatives’ experiences, their dilemmas, as well as significant factors in SDM processes with persons with intellectual disability. The objective was to obtain an understanding from relatives about what kinds of knowledge are needed for SDM processes and what challenges exist in practice. Such knowledge can provide important insight for service providers and other relatives. To this end, the article aims to answer the following research questions:

◦ What experiences do relatives of persons with intellectual disabilities have with supported decision making?
◦ Which dilemmas emerge in the supported decision-making processes?
◦ What are important factors in the supported decision-making processes seen from the relative’s perspective?

## Theoretical perspectives

This study aims to illuminate how relatives support persons with intellectual disabilities in making everyday decisions. We analyze and discuss everyday SDM processes through two theoretical lenses: Eva Feder Kittay’s care ethics and the theory of choice architecture, as developed by Richard Thaler and Cass Sunstein.

In *Learning from My Daughter,* Kittay (2019) develops an understanding of an ethics of care that involves both an attitudinal and an action component. She argues that caring requires more than concern about someone (attitude); it requires that one actually does something (action). Empirically, this means that care not only concerns meeting an individual’s basic needs but also promoting their flourishing and their best interests, in accordance with their own perspectives. Following Vehmas and Mietola (2021), we posit in this article that approaches solely centered on fulfilling basic needs frequently lead to an everyday life in which the individual’s personality, desires, and preferences are relegated to the background. Consequently, we employ care theory to scrutinize various support practices.

However, care is insufficient for developing further or understanding more comprehensively SDM. Decision-making is about making choices, which, for people with intellectual disabilities, often requires relatives’ creation of well-adapted frameworks that account for the person’s wishes and preferences. Therefore, the article also draws on Thaler and Sunstein’s (2019: p. 13) concept of choice architecture, which they define as “organizing the context in which people make decisions” with an intention to influence these decisions indirectly. When support persons “look after,” “facilitate,” or “regulate,” we use this perspective to analyze relatives support as support in making choices, thus as “choice architects.” Thaler and Sunstein link the term “choice architecture” to what they call libertarian paternalism—a philosophical argument that people should be free to make their own decisions but that it is also acceptable to influence their choices if this contributes to a healthier, better, and longer life for them. In this article, we do not limit the concept of choice architecture to libertarian paternalism, as Thaler and Sunstein’s argument assumes decision makers’ autonomy in a way that does not reflect persons with intellectual disability.

Although persons with intellectual disabilities belong to a heterogeneous group, many people have need for individual services and support through their adult everyday lives. Thaler, Sunstein and Balz argue that ‘Decision makers do not make choices in a vacuum. They make them in an environment where many features, noticed and unnoticed, can influence their decisions’ (Thaler et al., 2010). We expand their notion of environmental features to include care and social welfare service systems. Hence, we see such institutional systems as a contextual aspect of choice architecture. While we focus on the everyday support practices of relatives who indirectly and significantly influence the decisions of persons with intellectual disability, we also emphasize that this kind of contextual understanding is necessary. It should be mentioned that, while the theory of choice architecture has been utilized in various areas, including welfare and economic systems, it has not yet been utilized in the context of the lives of persons with intellectual disabilities. A more contextual understanding of choice architecture, which accounts for limited autonomy for both decision-makers and choice architects, will hopefully also increase the utility of Thaler and Sunstein’s work.

## Method

We conducted qualitative, in-depth interviews with relatives of adults with intellectual disability who live in municipal housing. The purpose was to gain access to detailed descriptions of relatives’ experiences and understandings of SDM processes. We chose individual interviews to obtain detailed information about relatives’ practices and thoughts on supported decision making.

### Research context

The informants in this study were relatives of adults with intellectual disabilities. These adults had different degrees of intellectual disability (mild to severe) and different ages (20 to 65 years), and they lived in municipal group homes over different periods. Typical of the Norwegian context, they lived in individual apartments within group homes. Hence, the daily life is characterized by some degree of private space, but these spaces are also sites of professional work. So-called housing leaders supervise the physical spaces, while also coordinating services, leading to what might be considered a hybrid context. This hybridity also pertains to the position of the CRPD in the Norwegian context since Norway has ratified but has yet to incorporate the convention. On the one hand, the ratification implies that services are obliged to provide SDM. On the other hand, the lacking incorporation means that neither people with intellectual disabilities nor their relatives can demand or seek enforcement of their right to SDM (Skarstad, 2023).

### Sample and interviews

Five of the informants were recruited via housing leaders in a specific county. We asked housing leaders to forward the information letter to the relatives of persons living in municipal housing. Relatives who wanted to participate then contacted us and a place and time for the interview was agreed upon. We then recruited the remaining six informants via our professional network. In total, eleven relatives participated in the study, including three mothers, three fathers, three sisters, and one couple (a mother and father).

We used a thematic, semi-structured interview guide (Brinkmann and Kvale, 2015; Daly, 2007), with the main questions centered around the following themes: “support to decide for yourself in everyday and major decisions in life,” “collaboration with the individual on choices and decisions,” and “challenges in SDM processes.” The first author conducted all the interviews physically, either in an office or at the informants’ home, except for two that were conducted digitally (via Microsoft Teams) due to distance and COVID-19 restrictions. The interviews lasted between 50 and 100 minutes.

### Research ethics

The study was approved by the Norwegian Agency for Shared Services in Education and Research (Sikt, project number 461257). Both the collection and storage of the data have been carried out in line with Sikt’s research ethics guidelines. All informants received both written and verbal information about the study and the right to withdraw from it at any time without having to give a reason. The informants gave both oral and written consent to participate in the study. All information about study informants, and their relatives with intellectual disabilities was anonymized.

The study explores relatives’ experiences with SDM. Although people with intellectual disabilities did not participate in the interviews themselves, and their consent was not required, it was important to respect their views and their “indirect participation.” Hence, the first author asked the informants to inform their relatives with intellectual disabilities about the study’s purpose. In this way, they could tailor the information to the person’s capacity to understand and also remain sensitive to their views and experiences.

Due to the sensitive nature of the topic and the fact that persons with intellectual disability are considered a marginalized group, it was important that the relatives felt safe and comfortable during the interviews. Therefore, we enabled the informants to choose where the interviews would be conducted. The first author, who conducted the interviews, remained sensitive to the relatives’ different perspectives and the complexities of their work.

### Analysis

Although we used the term “supported decision making” in the information letter sent to prospective informants, the term was used less frequently during the interviews. Instead, because the concept of SDM is still relatively new in Norway (Demic et al., 2023; Demic and Gjermestad, 2021), we used phrases such as “support to make your own choices,” “support to decide for yourself,” or “support to make your own decisions.” Of the 11 informants, only 3 used the term “supported decision making” themselves.

Braun and Clarke’s (2022) thematic analysis guide our analytical method. We carried out the analysis in five phases, where the first phase was focused on openly reading all the transcribed interviews. In phase two, we did more systematic work with generating introductory codes. Phase three is characterized as an active and interpretive process with the organization of codes in introductory themes. We reviewed the preliminary introductory themes in phase four and developed the main themes. The main themes (see Table 1) form the basis for the findings presented in the article. At this point, we also analyzed content in the main themes through two theoretical lenses: Ethics of care (Kittay, 2019) and Choice Architecture (Thaler and Sunstein, 2019). In the last phase, we defined the main interpretations by interpreting the findings through theoretical lenses.

**Table 1.** From introductory codes to main interpretations.

Introductory codes (examples)	Main themes based on codes	Theoretical lenses	Main interpretations
- To support choices that are important to the person - To support the person’s decision even if they are not always sensible - To be seen as a fellow human being with equality	Recognizing the individual and what they care about	Ethics of care and Choice Architecture	Care as a basis for choice architecture Choice architecture as a dimension of care Care, choice architecture, and time
- Getting hold of the will of the individual - Decision-making processes based on facilitation and guidance - To guide and explain through practical examples	Dialogue that triggers the individual’s perspective		
- Danger of stereotypical everyday life if you don’t seek out new experiences - To dare to be inventive - You don’t know if you like it until you’ve tried	Dare to try something new		
- Decisions that are controlled and monitored - Ensure that the person’s choice is safeguarded in risk situations	Dilemmas in supported decision-making processes		
- Challenges in complex choices - When here-and-now decisions have negative implications for the person’s long-term goals - Decisions that are regulated and corrected - When consequence thinking is absent	Short- and long-term supported decision making		
- When cooperation with others opposes decision-making processes - Challenging always to have to deal with a third party when making important decisions - When supporting the person’s choice is not enough for the realization of decisions	Systemic conditions for supported decision making	Ethics of care and Choice Architecture	Care and choice architecture in institutional context

## Findings

Six main themes were identified in the experiences of relatives through the analysis processes: (a) recognizing the individual and what they care about, (b) dialogue that triggers the individual’s perspective, (c) dare to try something new, (d) dilemmas in SDM processes, (e) SDM in the short and long term, and (f) systemic conditions for SDM.

### Recognizing the individual and what they care about

All relatives pointed to the importance of recognizing and supporting decisions that are important to the persons with intellectual disability. Such decisions can concern school, leisure activities, travel, and other important decisions such as having a boyfriend or consuming a cider (that contains alcohol) on a Saturday night. Relatives linked the opportunity to make choices to human dignity. One relative said: “It is extremely important because it is about human dignity. If you don’t get to make any choices, who are you?” Relatives emphasized the importance of having in-depth knowledge of the person, their wishes, interests, and what matters to them. Several described the importance of recognizing the person’s decisions and what they care about, even if these do not necessarily reflect their relatives’ values and norms. One sister said:

He buys expensive radio-controlled cars and hides them. It is probably because someone has said: “It was stupid that you spent so much money on those things” or “What nonsense.” I think it is very important that we recognize who they are, what decisions they want to make, and what interests they have. Setting aside one’s own norms is important.

All the relatives believed it was important to know the person’s experiences and life history, to form a picture of who they are, their wishes, and what they value. One informant, who also had the role of financial guardian, spoke about the importance of recognizing her brother’s decisions, even if they may not be the most financially profitable. She explained that it is particularly important to support decisions that lead to experiences of dignity and humanity: “He could go and get a haircut for NOK 300, but he often goes and gets a haircut for NOK 600. But there he is respected and treated like a human being.” However, even if relatives set aside their own norms and values, the person’s freedom to spend their own money on what they want is not unlimited. Relatives’ narratives show that there is still a framework in place, in which relatives maintain an overview of the finances and set limits so that the person spends some but not all their money on leisure activities.

Care and choice architecture theories also describe emotional sensitivity concerning how relatives organize and facilitate choices. For example, one of the relatives disclosed how her brother’s previous experience of being at a funeral informed his wish for their mother to be buried in a coffin made out of light-colored wood. By listening to her brother’s opinion and inviting him to participate in the decision-making process around their mother’s funeral, the relative constructed and created an opportunity for involvement in significant decisions in her brother’s life: “And then she (their mother) was lying there (in a coffin), so he picks up the cloth and checks whether they have changed the coffin to a pale-wooden one. And they had. And then he touched her, hugged her, and was so happy.” Here, a space is created that would not have been possible without care but could also become overwhelming without choice architecture.

### Dialogue that triggers the individual’s perspective

All relatives recounted their strategies to identify the person’s wishes or perspectives when these were not explicitly expressed. Several described how they sought out the person’s wishes and preferences in each situation through tailored conversations. One relative talked about how, through a genuine interest in her brother’s opinion, she opens and frames a dialogue that can trigger his perspective and help him to stand up for his choices:

If he doesn’t want to visit, he has difficulty saying so. So, he chooses not to pick up the phone. It is something we have worked on a lot. It is very important for competence in making choices. We must learn that it is also okay to say no. We are allowed to say that we didn’t want to do this or that we didn’t bother. Sometimes, we don’t feel like it, which must be okay.

Some relatives explained that, sometimes, this kind of dialogue could lead to the person finally being able to respond “No,” when invited to visit’. All of the informants shared how demanding it can be to determine the person’s preferences and wishes concerning abstract things. One mother shared a story marked by insecurity and uncertainty regarding how to offer support in a particular situation. Despite her daughter’s preference not to receive suggestions, the mother draws on past experiences with her daughter’s SDM processes. This allows her to place her daughter in context and provide customized information about the choice at hand:

She never wants a suggestion. However, I can say that “We can do this or that.” And then you must be able to show it off, or it should be something you can grab onto. And when she realizes that she has a choice, she can say: “I don’t know. What do you think?” Then I can say: “I would have it or maybe do it like that.” And during such a process where I have shared my reflections around my decision-making process, she then can make a choice. She can also suddenly say: “I want it.” It depends on me bringing her into a context or someone else’s reflection on the same choice. Trigger a small decision-making process.

The relatives’ narratives show that, even when one knows the person well, eliciting their preference can be experienced as demanding —for both parties. The narratives reflect how being caring, considerate, and/or simply asking for the person’s opinion is insufficient in many everyday SDM situations. Informants explained how they had to learn to navigate an unclear terrain characterized by uncertainty. Several believed that, to facilitate and trigger the person’s perspective and opinion, they had to frame the SDM situation based on genuineness and familiarity, tailored to the individual. In this practice, care and choice architecture meet.

### Dare to try something new

The informants spoke about how venturing beyond established practices and norms can provide insights into the person’s preferences, shedding light on what they like and dislike. By daring to move beyond everyday routines and familiar and safe activities, several relatives felt that they could create space for new choices and improve the individual’s life. A father expressed that, although his daughter appeared satisfied with doing the same activity year-round at the day center, this does not mean she should not be presented with new alternatives:

I think she is happy. I am very dissatisfied! The problem is that she sits there and makes Christmas presents all year. There are no demands and no one to reach for. This is mostly not how we feel in life. When she is with us (parents), she initiates much more often activities.

In the above quote, we see the father’s frustration with his daughter’s restricted options for activity. We also see how he actively tries to facilitate opportunities that can expand her range of experiences and her mastery, throughout her life. Many of the relatives expressed that life can become monotonous if support persons do not take responsibility for organizing and creating space for new experiences that promote choices and reveal preferences. One father stated that, though his son never asks and rarely expresses that he wants to “participate in something,” he is a person who, if he is introduced to something, will “participate in most things”:

He's involved in most things, actually. If we’re to go for a sail, we just change our clothes and take life jackets. He has also been involved in snowmobiles and motorcycle sidecars. As long as he is with someone he trusts, whether it is a service provider or me, it does not matter. As long as you take it carefully, it goes very well. Often there is discouragement (in the municipal housing) regarding trying new things. He never expresses “Now I want to try it” or “Now I want to do it.” It doesn’t happen.

Relatives’ experiences show that SDM can be challenging in practice. Engaging in novel activities often involves pushing the individual beyond their comfort zone, with the aim of discovering and developing new preferences that could improve their life. While relatives acknowledge their duty to enrich the lives of persons with intellectual disability and determine their preferences, the narratives indicate that they sometimes lack control over the outcome when introducing new activities. Relatives’ actions point to the importance of providing care within a safe framework for exploring the person’s preferences.

### Dilemmas in supported decision-making processes

The relatives spoke about various dilemmas they encounter in the SDM processes. Several described challenging situations when they have tried to balance between the individual’s wishes and needs and the risk of consequences. One mother shared how, while she and her husband supported their daughter’s wish to have a boyfriend, they were concerned about the way their daughter sought out potential boyfriends:

We still don’t know how my daughter met this boy. She probably only sent friend requests on Facebook because she was on a very intense hunt for a boyfriend for a while. She also met him in one way or another. We are very clear that we want her to be well. We understand her desire to have a boyfriend, but we have been very clear that her method of finding a boyfriend—to add people online arbitrarily—is not right. It’s terrifying.

Relatives acknowledged the importance of people with intellectual disabilities being able to make decisions about their own lives; at the same time, they expressed that it was their responsibility to look after the persons’ basic needs and safety. In the interviews, informants noted the challenging nature of caregiving situations, emphasizing the heightened tension associated with making decisions that involve risk. Their narratives reveal that, despite granting persons with intellectual disabilities the freedom to act based on their own wishes and preferences, such autonomy exists within specified frameworks where relatives maintain constant oversight. Even in their attempts to prevent negative consequences, the relatives’ experiences highlight their persistent feelings of worry, doubt, and uncertainty, as they lack control over potential outcomes.

Some of the relatives spoke about being faced with the following dilemma: What if the person’s preferences in each situation brought joy but also greater long-term health challenges? One mother described how, on the one hand, her daughter’s compulsive behavior of collecting things improves her life, as it enables her to access information about various events in which she is interested; on the other hand, it can negatively affect her mental health:

She is very good at picking up brochures. And they (municipal housing residents) receive mail about adapted offers. And she collects that. She has a lot of lists and collects advertisements and brochures, and that is also a problem. She can quickly become a bit of a bag lady. There is so much junk. But she reads this and ticks off things she wants to participate in. So, this weekend, she took part in three flea markets because she had received the information about them.

The dilemma with which this mother grapples illustrates how a single decision has the potential to simultaneously enrich and impede her daughter’s life. In this instance, the mother opts for limiting her intervention. She prioritizes what brings joy to her daughter, recognizing that constraining this freedom to gather diverse information would limit her daughters access to arenas that bring her joy. The relatives’ experiences can thus be understood as navigating and accepting a certain level of risk, which, while challenging, is also acknowledged as a natural facet of life for all individuals.

### Short- and long-term supported decision making

Both professional and caregiver support extend beyond immediate assistance with decision making; it also concerns the effect of decisions in the longer term. One relative described the challenges that arise due to her brother’s refusal to be vaccinated against COVID-19, directly influencing his goal to travel to Spain: “He loves traveling to Spain, those are his highlights. He probably does that four to five times a year with an uncle.” She, like other informants, pointed to challenges that arise in complex decision making, where the person with intellectual disability is unable to translate their immediate choices into future consequences: “He doesn’t see that what he does now affects what happens tomorrow because that long-term thinking is mostly absent.” This relative spoke about how she uses persuasion in the here-and-now to ensure that her brother can achieve his long-term goal of going to Spain.

The informants highlighted many of these kinds of dilemmas. Their experiences point to uncertainty around where to draw a line between supporting versus overriding the person’s choices and preferences. For example, one mother explained how she must choose to override her daughter’s preference to wear winter clothes when it is warm outside:

She wears winter shoes and a puffer jacket now, in spring. She would probably do that all summer. In practice, the solution is that the next time I visit her, I say: “You don’t have much space. I returned your summer shoes—now it’s summer. I’ll take the winter shoes home with me and look after them there until winter comes.” I think I do it as a mother. But it is at the intersection of when this becomes coercion.

The story shows how demanding it can be to be in, supported decision-making processes. On the one hand, one can argue that the mother would provide a fair choice architecture if she would avoid all interference letting her daughter follow her own wishes and preferences in the here-and-now. In that case the mother could choose to have her daughter’s winter clothes easily available in the wardrobe in the summer, the disadvantage of that strategy being that her daughter might get too hot in summertime, negatively affecting her overall quality of life.

On the other hand, placing winter clothes out of sight may improve the daughter’s overall quality of life. This is the strategy of which the mother’s narrative speak. Yet this strategy can be understood as intrusive and paternalistic: What will be her daughter’s preferences when she encounters major challenges with seasonal transitions every year? Should her daughter’s objection to changing seasonal clothes be interpreted as her preference? Or can one, as the mother does, build on previous experiences and interpret the transition to summer as a stressful situation - in which her daughter needs help to form her preferences preferably from someone who cares and frames choices for her?

Adding to this complexity, a distinction between short- and long-term goals is pertinent in this dilemma. The mother is also using her intimate knowledge to help the daughter achieve her long-term goal: to participate in and benefit from activities she prefers and enjoys in everyday life. Thus, care and choice architecture are not only interwoven to make short-term decisions but also long-term.

### Systemic conditions for supported decision making

The informants highlighted that SDM situations are more complex than others. They explained various professionals who might or might not know the individual well – not only in the group homes, but also in social services administration, day centers, and so on – nevertheless had strong opinions about the best choice for them. Hence, the actual SDM process was bound up with complex relations between many parties. Relatives described also how systems and frameworks with which persons with intellectual disability must deal create challenges and are perceived as inhibiting factors in SDM processes. As one sister expressed, “He (her brother) is rarely allowed to make decisions about where he wants to live, what he wants to do, how will he work without another party or a framework that we have to deal with.” The informants’ experiences show how obtaining housing, a job, daycare facilities, or other services based on the person’s needs and preferences is not a given, and that it is often difficult to achieve this. One relative shared:

He was moved from regular to supported employment because he often experienced headaches. But he had long wanted to return to regular employment where he had previously worked. Getting such an arrangement back has been difficult because it is not desirable for NAV (The Norwegian Labor and Welfare Administration). But now he has a job at a grocery store three days a week. We feel that being back in regular employment is very good for his mental health.

Relatives shared that they have had to learn about the barriers persons with intellectual disability face when trying to reclaim their employment after an extended illness. Relatives describe these barriers as stereotypical systems where all persons with intellectual disabilities, regardless of their functioning, preferences, and needs, are commonly offered a job in supported employment. The encounter with such prejudiced systems often includes having to convince state agencies of their ability to handle a job they previously held. In such situations, institutional bureaucratic conditions were not the only limiting SDM processes those relatives experienced. Lack of familiarity with the person was also a limiting factor, especially considering how it affects the organization of support and assistance from public agents. The informants’ suggest that the relatives’ commitment to supporting the person from the person’s perspective, their care, and in-depth knowledge of the person were necessary for the person’s opinions, wishes, and preferences to be in focus in such complex SDM processes. All the relatives referenced experiences similar to the following examples, provided by two informants:

He must consider whether the municipality says yes or no. He must consider for how many hours he will receive assistance to attend leisure activities. He has long wanted to start e-sports, but no one supported him. That’s what I think is challenging about the municipality saying that he should be allowed to decide. But then there are so many factors that make it impossible.

He didn’t go to a daycare center during the COVID-19 lockdowns and was therefore very frustrated. He loves going to this daycare center job. Can’t say it strongly enough. So, when the neighboring municipality maintained the service while our municipality shut it down during the COVID-19 lockdown, it was a slap in the face.

These micro-narratives show that many things in the environment can impact decisions. Relatives experience institutional frameworks and financial conditions integrated into such choice architectures as inhibiting factors/conditions regarding SDM. Several relatives described reaching a point where they decided to abandon the pursuit after enduring lengthy bureaucratic processes. Existing frameworks are portrayed as working against them, rendering it impossible to reach a decision that aligns with the person’s preferences.

## Discussion

Self-determination is often described as something persons with intellectual disability can achieve or realize. As such, self-determination is described using psychological theories about individual freedom of action (Wehmeyer and Little, 2015). SDM, on the other hand, refers to active interaction processes, in which persons are enabled to exercise their legal capacity to act (Browning et al., 2014). Findings from the present study show that both choice architecture and care are useful concepts for understanding relatives’ contributions to these processes. In the following, we discuss this in more detail and also argue for a broader understanding of these concepts and their correlation.

### Care as a basis for choice architecture

The study’s findings show that relatives can be seen as both caregivers and choice architects who, through structures, frameworks, and support, facilitate persons with intellectual disability to express their preferences and make decisions that reflect their perspective. Although the values of the relatives in our study do not always match the values of the person they support, relatives’ attitudes and actions can be interpreted as appreciating and in line with the preferences of persons with intellectual disability. In this way, relatives also demonstrate the importance of reflection and awareness of their own values and their possible impact on SDM processes.

However, findings also show that this freedom to make decisions in line with one’s own preferences is not unlimited. Caregivers are still responsible for ensuring that the person has enough money to cover basic needs and that not all monthly income is spent on, for example, radio-controlled cars and other interests. Our findings clarify how relatives’ careful support and knowledge of the person’s preferences are important when they, as choice architects, organize frameworks within which persons with intellectual disability can decide for themselves.

This study’s findings support Kittay’s (2019) understanding of the importance of subjective desires for flourishing and providing care that emphasizes not only basic needs but also one’s likes and preferences. In the interviews, relatives underscored the significance of fostering the person’s involvement. They established a secure framework for exploring new activities, recognizing that some persons with intellectual disabilities may never explicitly express a desire to try something new—to say “Now I want to try it.” This study’s findings suggest that it is not enough for support persons to care for and acknowledge the person being supported, but they must also actively act (Kittay, 2019) and create frameworks (Thaler and Sunstein, 2019) for exploring “the new,” in order to promote self-determination in the long term. These findings are supported by research that highlights the importance of encouragement and opportunities to experience different environments, activities, and people throughout life, as this promotes awareness of one’s preferences, life alternatives, and opportunities to learn and act autonomously (Curryer et al., 2015; Shogren, 2020; Vehmas and Mietola, 2021).

### Choice architecture as a dimension of care

In the relatives’ narratives, we can see many instances in which they demonstrate respect for the person’s dignity and interpersonal contact by offering options from which the person with intellectual disability can choose: for example, with regards to a hairdresser or shop. While the relatives (as well as the shop workers and hairdressers) can be viewed as persons who care about and recognize the citizenship of persons with intellectual disability, they can also be viewed as facilitators or choice architects (Thaler and Sunstein, 2019) who can provide support in many SDM processes. One example is how the relatives in this study show care by creating spaces for and facilitating new activities. In this way, they contribute to the person’s exploration of new experiences, helping them participate in activities in which they might otherwise have never considered participating (Thaler and Sunstein, 2019). Moreover, this helps the relatives broaden their knowledge about the person’s preferences.

Correspondingly, the relatives’ experiences—as related in the interviews—show that, even if persons with intellectual disability are aware of their own preferences, they may require support to express those preferences. Indeed, their preferences and goals may sometimes require a caring interaction, such as self-designed genuine dialogue and customized information. Our findings suggest that close, relationships can be crucial for effective SDM (Agran et al., 2010; Knox et al., 2017). Hence, these relationships can be called caring relationships.

### Care, choice architecture, and time

Kittay’s (2019) theory of care prioritizes relationships, particularly strong moments in these relationships. However, our findings indicate that SDM processes depend on preferences actually being discovered, expressed, and respected. Our analysis suggests that it cannot be assumed that all persons with intellectual disability know their preferences in each situation. The degree of support they receive in exploring new activities is therefore crucial for their capacity to develop preferences—and then in the long term, the ability to make decisions based on those preferences. This underscores the importance of supportive environments for the development of decision-making skills in SDM processes (Shogren, 2020).

The relatives’ experiences with complex decisions reveal the dilemmas they face, when confronted with the need to balance supporting the person’s immediate preferences with longer-term goals. An example of this is the mother whose daughter signaled her reluctance to wear seasonally appropriate clothes. On the one hand, if the mother immediately acquiesces, this could be seen as a short-term solution that might have negative consequences for her daughter in the long run. On the other hand, when the mother chooses to intervene and remove the winter clothes for summer, despite her daughter’s verbal resistance, this reflects a decision grounded in her knowledge of her daughter and what induces stress and frustration for her daughter in situations of transition.

The dilemmas that often arise in particularly challenging everyday SDM situations, where the consequences of the choices are serious, indicate that we should conceptualize SDM as an interweaving of short-term and long-term support. Relatives’ experiences show that caring for and focusing on the person’s right to self-determination can necessitate limited choice options in complex decisions. Likewise, limiting one’s environment or choices may serve the person’s long-term goal and make them more capable of becoming independent in the long run. Since these findings bring forth an intertwining of care and facilitating for every day decision making, they can also be interpreted in light of Skarstad’s (2023: p. 16) argument that it might be advisable to present fewer choices to persons with intellectual disability, to help them better realize their self-determination and reach their long-term goals or “the goal that the person greatly value”.

### Care and choice architecture in institutional context

Kittay’s concept of an ethics of care (2019) is productive for understanding this study’s findings. Simultaneously, we note that Kittay’s concept does not point to institutional conditions that can be limiting in such everyday situations. Our study shows that institutional conditions significantly affect both opportunities for SDM and the outcome of important decisions (such as work) for persons with intellectual disabilities. Caring for relatives therefore involves supporting the person’s wishes and preferences in the face of limitations at a structural level.

Similarly, Thaler and Sunstein have been criticized for presupposing autonomy and free actors (Engelen and Nys, 2020) which requires greater independence than many persons with intellectual disability have. On the one hand, many choices are complex, in line with Thaler and Sunstein’s (2019) description of rare and difficult choices that are well-suited to a choice architecture conceptual lens. On the other hand, the findings in our study also emphasize the importance of the role of carers. In the absence of close relationships, the choice architects will often be persons who do not know the person well; they may restrict the opportunity for a person with intellectual disability to participate in decision making about their own life. In the same way, the role of relatives is particularly important in such complex relationships and processes.

SDM processes in this study can be understood in the light of support persons’ commitment role (Bigby and Douglas, 2020) to initiate meetings and act in ways that can lead to the realization of the person’s preferences and wishes regarding employment or health and care services. In our study, several relatives highlighted experiences where institutional frameworks were limiting conditions in SDM processes. The system, they noted, tends to disregard the person’s own preferences, and often expects persons with intellectual disabilities to conform to existing frameworks. This is evidenced in a recent political document ‘Norwegian national guidelines for health care services provision’ that claims many persons with intellectual disabilities in Norway are ‘outside ordinary work, despite the fact that there are many of them who could have had ordinary work, possibly with individual accommodation’ (Helsedirektoratet, 2021: p. 39). Undoubtedly, such institutional choice architecture, which takes little account of individual preferences, undermines SDM and the realization of self-determination.

## Conclusion

It is often assumed that persons with intellectual disabilities are unable to safeguard their self-determination without others’ help and support (Wehmeyer and Little 2015). The study demonstrates the complexity of SDM in the lives of persons with intellectual disabilities. By including the relatives’ experiences, we aimed to enrich the understanding of such complex decision-making processes.

In analyzing narratives about SDM, the analytical concepts of care ethics and choice architecture both proved illuminating and fruitful. Empirically, the study shows that: (a) supported decision-making sometimes requires careful facilitation with a focus on the person's preferences/perspective; this careful facilitation can be understood in terms of choice architecture, (b) choice architecture in the context of intellectual disability requires an intentionality of care and a focus on the person’s preferences/perspective, (c) relatives are concerned, not only with support in the moment, but also the effect of the decision in the long term, and (d) relatives’ care is a significant factor in meeting choice architecture in systemic conditions.

This article suggests that an ethics of care without action, might not reflect the reality of these relatives. Providing choice architecture as a form of action, without the correct intentionality to provide care and capture the person’s goals, wishes, and preferences in the short and long term is also not the way to go. In discussions about how to provide SDM, active structuring of the context to influence the person with intellectual disability is acceptable, as long as the relatives themselves constantly reflect that the person’s preferences are at the center of the decision.

The study brings forth an inextricable relationship between care ethics and choice architecture in SDM practices. Given that self-determination is also a political goal, in line with the CRPD, this is also a political matter. In demonstrating that neither freedom of choice nor care are sufficient, the study also has implications for policies and services. If self-determination is promoted by the synergy between choice architecture and care, not least in the long term, relatives’ experiences are also politically important.

Finally, the study offers a unique theoretical contribution reflected in bringing together Kittay’s ethics of care and Thaler and Sunstein’s choice architecture. While the two concepts were rarely referenced together, we hope that the dynamics will prove fruitful for other research frontiers.

## References

[bibr1-17446295241297184] Agran M Storey K Krupp M (2010) Choosing and choice making are not the same: Asking ‘what do you want for lunch?’ is not self-determination. Journal of Vocational Rehabilitation 33(2): 77-88. 10.3233/JVR-2010-0517

[bibr2-17446295241297184] Demic S Gjermestad A (2021) Å stå i det uvisse. Tjenesteyteres erfaringer med beslutningsstøtte i møte med personer med alvorlig utviklingshemming i kommunale botilbud. Tidsskrift for omsorgsforskning 7(1): 1-15. 10.18261/issn.2387-5984-2021-01-04

[bibr3-17446295241297184] Demic S van den Breemer R Hanisch H , et al. (2023) Beslutningsstøtte i kommunale botilbud for personer med utviklingshemming. Fontene forskning 16(1): 4-19. https://hdl.handle.net/11250/3118580

[bibr4-17446295241297184] Braun V Clarke V (2022) Thematic analysis. A practical guide. London: Sage.

[bibr5-17446295241297184] Brinkmann S Kvale S (2015) InterViews: Learning the Craft of Qualitative Research interviewing. Thousand Oaks: Sage Publication.

[bibr6-17446295241297184] Bigby C Whiteside M Douglas J (2019) Providing support for decision making to adults with intellectual disability: Perspectives of family members and workers in disability support services. Journal of Intellectual & Developmental Disability 44(4): 396–409. 10.3109/13668250.2017.1378873

[bibr7-17446295241297184] Bigby C Douglas J (2020) Supported decision making. In Stancliffe RJ Wehmeyer ML Shogren KA , et al. (eds) Choice, Preference, and Disability: Promoting Self-Determination Across the Lifespan. Cham: Springer, pp. 45–66.

[bibr8-17446295241297184] Blanck P Martinis JG (2015) The right to make choices: The national resource center for supported decision-making. Inclusion 3(1): 24–33. 10.1352/2326-6988-3.1.24

[bibr9-17446295241297184] Bowey L McGlaughlin A (2005) Assessing the barriers to achieving genuine housing choice for adults with a learning disability: The views of family carers and professionals. British Journal of Social Work 35(1): 139–148. 10.1093/bjsw/bch167

[bibr10-17446295241297184] Browning M Bigby C Douglas J (2014) Supported Decision Making: Understanding How its Conceptual Link to Legal Capacity is Influencing the Development of Practice. Research and Practice in Intellectual and Developmental Disabilities 1(1): 34-45. 10.1080/23297018.2014.902726

[bibr11-17446295241297184] Curryer B Stancliffe RJ Dew A (2015) Self-determination: Adults with intellectual disability and their family. Journal of Intellectual and Developmental Disability 40(4): 394-399. 10.3109/13668250.2015.1029883

[bibr12-17446295241297184] Daly KJ (2007) Qualitative Methods for Family Studies & Human Development. Los Angeles: Sage Publications.

[bibr13-17446295241297184] Engelen B Nys T (2020) Nudging and autonomy: Analyzing and alleviating the worries. Review of Philosophy and Psychology 11(1): 137-156. 10.1007/s13164-019-00450-z

[bibr14-17446295241297184] Helsedirektoratet (2021) Gode helse- og omsorgstjenester til personer med utviklingshemming. Nasjonal veileder. https://www.helsedirektoratet.no/veiledere/gode-helse-og-omsorgstjenester-til-personer-med-utviklingshemming

[bibr15-17446295241297184] Kittay EF (2019) Learning from my Daughter: The Value and Care of Disabled Minds. New York: Oxford University Press.

[bibr16-17446295241297184] Knox L Douglas JM Bigby C (2017) ‘I’ve never been a yes person’: Decision-making participation and self-conceptualization after severe traumatic brain injury. Disability and Rehabilitation 39(22): 2250-2260. 10.1080/09638288.2016.1219925 27547914

[bibr17-17446295241297184] NOU 2019: 14 (2019) Tvangsbegrensningsloven – Forslag til felles regler om tvang og inngrep uten samtykke i helse- og omsorgstjenesten. Oslo: Helse- og omsorgsdepartementet. https://www.regjeringen.no/no/dokumenter/nou-2019-14/id2654803/.

[bibr18-17446295241297184] Seale J Nind M Simmons B (2013) Transforming positive risk-taking practices: the possibilities of creativity and resilience in learning disability contexts. Scandinavian Journal of Disability Research 15(3), 10.1080/15017419.2012.703967

[bibr19-17446295241297184] Shogren KA (2020) Self-Determination, Preference, and Choice. In Stancliffe RJ Wehmeyer ML Shogren KA , et al. (eds) Choice, Preference, and Disability: Promoting Self-Determination Across the Lifespan. Cham: Springer, pp.27–43.

[bibr20-17446295241297184] Skarstad K (2023) Oppression or Support? Social Policy in the Lives of Persons with Intellectual Disabilities. Nordic Journal of Human Rights 41(4): 393–409. 10.1080/18918131.2023.2197721

[bibr21-17446295241297184] Thaler RH Sunstein CR (2019) Nudge – Hvordan ta bedre valg om helse, penger og lykke. Oslo: Dreyers forlag.

[bibr22-17446295241297184] Thaler RH Sunstein CR Balz JP (2010) Choice architecture (SSRN Working Paper Series No. 1583509). Retrieved from the Social Science Research Network website: 10.2139/ssrn.1583509

[bibr23-17446295241297184] Then SN Carney T Bigby C , et al. (2018) Supporting decision-making of adults with cognitive disabilities: The role of Law Reform Agencies – Recommendations, rationales and influence. International Journal of Law and Psychiatry 61, 64–75. 10.1016/j.ijlp.2018.09.001 30245192

[bibr24-17446295241297184] United Nations (2006) Convention on the Rights of Persons with Disabilities (CRPD). *Available at* : https://www.un.org/development/desa/disabilities/convention-on-the-rights-of-persons-with-disabilities.html (accessed 1 March 2023). 10.1515/9783110208856.203 18348362

[bibr25-17446295241297184] Vehmas S Mietola R (2021) Narrowed Lives: Meaning, Moral Value, and Profound Intellectual Disability. Stockholm University Press.

[bibr33-17446295241297184] Watson J Wilson E Hagiliassis N , et al. (2017) Supporting end of life decision making: Case studies of relational closeness in supported decision making for people with severe or profound intellectual disability. Journal of Applied Research in Intellectual Disabilities 30|(6): 1022–1034. doi: 10.1111/jar.12393 28815814

[bibr26-17446295241297184] Wehmeyer ML Little TD (2015) Self-Determination. In Wehmeyer ML (ed) The Oxford Handbook of Positive Psychology and Disability. New York: Oxford University Press, pp.116-136.

[bibr27-17446295241297184] Wiesel I Bigby C Kamstra P , et al. (2021) Possibility and risk in encounters between people with and without intellectual disability. Journal of Intellectual & Developmental Disability 46(1): 35–44. 10.3109/13668250.2020.1837606 39818588

